# A novel method for estimating the strength of positive mating preference by similarity in the wild

**DOI:** 10.1002/ece3.2835

**Published:** 2017-03-22

**Authors:** Mónica Fernández‐Meirama, Daniel Estévez, Terence P. T. Ng, Gray A. Williams, Antonio Carvajal‐Rodríguez, Emilio Rolán‐Alvarez

**Affiliations:** ^1^ Departamento de Bioquímica, Genética e InmunologíaFacultad de BiologíaUniversidad de VigoVigoSpain; ^2^ School of Biological SciencesThe Swire Institute of Marine ScienceThe University of Hong KongHong Kong SARChina

**Keywords:** assortative mating, correlation coefficient, mate choice, mating pairs, scale‐of‐choice effect

## Abstract

Mating preference can be a driver of sexual selection and assortative mating and is, therefore, a key element in evolutionary dynamics. Positive mating preference by similarity is the tendency for the choosy individual to select a mate which possesses a similar variant of a trait. Such preference can be modelled using Gaussian‐like mathematical functions that describe the strength of preference, but such functions cannot be applied to empirical data collected from the field. As a result, traditionally, mating preference is indirectly estimated by the degree of assortative mating (using Pearson's correlation coefficient, *r*) in wild captured mating pairs. Unfortunately, *r* and similar coefficients are often biased due to the fact that different variants of a given trait are nonrandomly distributed in the wild, and pooling of mating pairs from such heterogeneous samples may lead to “false–positive” results, termed “the scale‐of‐choice effect” (SCE). Here we provide two new estimators of mating preference (*C*_rough_ and *C*_scaled_) derived from Gaussian‐like functions which can be applied to empirical data. Computer simulations demonstrated that *r* coefficient showed robust estimations properties of mating preference but it was severely affected by SCE,* C*_rough_ showed reasonable estimation properties and it was little affected by SCE, while *C*_scaled_ showed the best properties at infinite sample sizes and it was not affected by SCE but failed at biological sample sizes. We recommend using *C*_rough_ combined with the *r* coefficient to infer mating preference in future empirical studies.

## 1 Introduction

Individuals of many animal taxa display mating preferences (Andersson, 1994) which can be defined as the sensory and behavioral properties that affect the propensity of individuals to mate with particular phenotypes (Heisler et al., 1987; Jennions & Petrie, 1997). Mating preferences often, however, incur some fitness costs such as increased time, energy expenditure, and predation risk during the search for mates. As a result, the mechanism by which mating preference may evolve remains the subject of controversy (Clark et al., 2007; Gavrilets, 2004). A range of potential benefits of exhibiting a mating preference which may outweigh these costs has been proposed, such as improved paternal care or the acquisition of “better” genes from mating with “high‐quality” mates (Andersson, 1994), and avoiding inbreeding (Consuegra & Garcia de Leaniz, 2008; Landry, Garant, Duchesne, & Bernatchez, 2001; Lumley et al., 2015; Yeates et al., 2009). Mating preference may also evolve through incidental runaway (sexual) selection (Chandler, Ofria, & Dworkin, 2013; Lande, 1981), sexual conflict (Arnqvist, Rowe, Krupa, & Sih, 1996), or by other mechanisms (Blyton, Shaw, Peakall, Lindenmayer, & Banks, 2016; Eddy et al., 2016; Hine, McGuigan, & Blows, 2011).

Irrespective of the evolutionary causes, exhibiting a mating preference has two distinct key evolutionary consequences (Gavrilets, 2004; Lewontin, Kirk, & Crow, 1968; Merrell, 1950): sexual selection (changing the probability of transmitting alleles in progeny of the preference‐targeted trait, sensu Arnold & Wade, 1984) and assortative mating (nonrandom mating between individuals bearing different phenotypes/genotypes, Gavrilets, 2004). These processes can be linked in certain scenarios, as a preference causing positive assortative mating (similar types are more frequent in mates than expected by chance) is expected to generate positive frequency‐dependent sexual selection (Servedio, 2016), while a preference causing negative assortative mating (different types are preferred in mates) is expected to produce negative frequency‐dependent sexual selection (Pusey & Wolf, 1996; Takahashi & Hori, 2008). These concepts and their definitions have, however, been the subject of much debate over the past decades (Arnold & Wade, 1984; reviewed in Andersson, 1994; Edward, 2015; Gavrilets, 2004).

Mating preference has been shown to play a key influence in the theoretical dynamics of several evolutionary processes: assortative mating and sexual selection (consequences of mating preference), for example, may contribute to reproductive isolation between incipient taxa (Gavrilets, 2004, 2014; Santos, Matos, & Varela, 2014; Servedio, 2016; Thibert‐Plante & Gavrilets, 2013; Thibert‐Plante & Hendry, 2011; Turelli, Barton, & Coyne, 2001). A major question is whether the theoretical conditions that allow the evolution of mating preference (intermediate levels of disruptive selection, low mating cost, strength of the mating preference, etc., see Gavrilets, 2004) in sympatry can be empirically observed in the wild. A difficulty in answering this question remains in linking theoretical arguments (and definitions) with empirical estimates (Gavrilets, 2004; Servedio, 2016; but see Roff & Fairbairn, 2015 for an exception). The methods to model mating preference and their consequences (e.g., assortative mating) have, however, not been empirically validated. To attempt to address this we briefly review the main strategies to model theoretically, and estimate empirically, true mating preferences from field data in an attempt to integrate these approaches.

Two mating preference mechanisms have been modelled depending on the evolutionary scenario considered (reviewed in Gavrilets, 2004; Kirkpatrick, Rand, & Ryan, 2006; Servedio, 2016; and ignoring any indirect mechanism to find a mate via habitat choice, resource search, etc.). The first mechanism refers to the case where individuals of the choosy sex (usually females) prefer certain mates that display particular variants of a trait (see Gavrilets, 2004). Such form of mating preference may lead to sexual selection and, hence, as a strong driver of extreme sexual dimorphism (e.g., weapons and ornaments in one sex but not in the other) observed in many birds and insects (Crespi, 1989; Futuyma, 2013). The second is a preference based on phenotype matching or similarity (i.e., a tendency to choose mates possessing similar variants of a trait), and such a preference by similarity may lead to positive assortative mating observed in many species (reviewed in Arnqvist et al., 1996; Crespi, 1989; Jiang, Bolnick, & Kirkpatrick, 2013; Servedio, 2016). These preferences can be modelled by using explicit genetic mechanisms (Kirkpatrick et al., 2006; Servedio, 2016) or by Gaussian‐like mathematical functions (Gavrilets, 2004, 2014; Lande, 1981). Explicit genetic mechanisms are often adequate to model the effects on qualitative traits (e.g., color) assuming one or two loci control the mating preference, while Gaussian‐like functions seem more appropriate to model quantitative trait loci (e.g., size and length, Lande, 1981; Roff & Fairbairn, 2015). For example, under a positive preference by similarity, any preference function should give a higher probability of mating when the mating individuals share similar variants of a trait (e.g., similar color or size, Carvajal‐Rodríguez & Rolán‐Alvarez, 2014).

Traditionally, the Gaussian‐like functions originally developed for theoretical studies were not, however, applicable to empirical data but recent modifications now allow their application (Carvajal‐Rodríguez & Rolán‐Alvarez, 2014). Different strategies have been considered to infer mating preferences empirically. Laboratory choice experiments, for example, have been used to investigate the mechanisms of mating preference (Coyne, Elwyn, & Rolán‐Alvarez, 2005; Knoppien, 1985), and the associated statistical tools to analyze such experiments have also been developed (Gilbert & Starmer, 1985; Rolán‐Alvarez & Caballero, 2000). These approaches, however, have limitations because mating is often difficult to induce under laboratory conditions, and the patterns observed under such conditions may not reflect the true mating patterns which occur in the field (Coyne, Kim, Chang, Lachaise, & Elwyn, 2002; Coyne et al., 2005).

**Figure 1:**
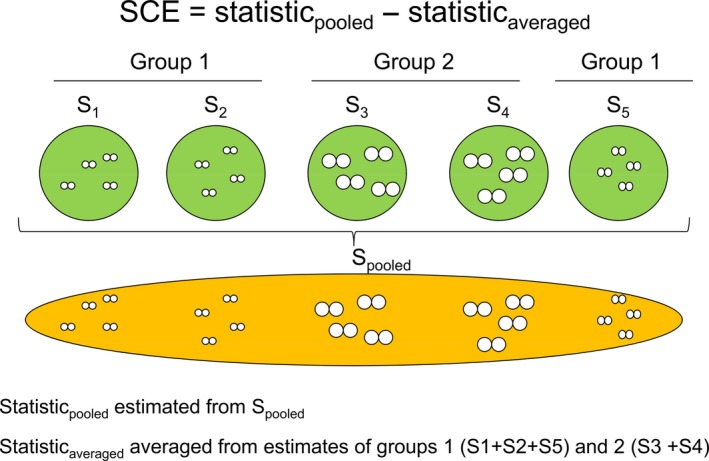
Scheme to explain how scale‐of‐choice effect (SCE) bias is estimated (modified from Ng et al., 2016). The small, white, circles in pairs represent putative mating pairs, while the relative size of these circles is correlated with the trait mean. The SCE occurs as a consequence of pooling mating pairs at a larger scale (*S*_pooled_; yellow area), while mate choice is actually produced at a smaller scale (within S1–S5; green areas), and in addition, there are some trait heterogeneity at this scale (between S1 to S5). Therefore, a way to estimate the SCE is to measure the statistic (Pearson's *r*,* C*_scaled_, or *C*_rough_) at the pooled level minus the average value within homogeneous groups (Groups 1 and 2). Note that SCE is expressed in the same units than the statistic used

An alternative strategy is to measure the strength of mating preference by observing mating pairs directly in the field (reviewed in Crespi, 1989; Jiang et al., 2013). In this second strategy, there is one statistical tool (PSI; the ratio of the observed frequency of a pair/expected frequency under random mating; see Rolán‐Alvarez & Caballero, 2000) available that could, under certain scenarios, estimate mating preferences for qualitative traits (e.g., color) in the wild. There is, however, no direct estimator of mating preference for quantitative traits (e.g., size) in the wild. Therefore, most authors have adopted an indirect approach for estimating the mating preference, focusing either on assortative mating or on sexual selection effects. When estimating assortative mating in the field (presumably caused by mating preference by similarity), the most common strategy is to use the Pearson's correlation coefficient (*r*) or related statistics on the trait values across the range of observed mates (reviewed in Jiang et al., 2013); the larger the coefficient, the stronger the preference by similarity. Recently, however, it has been shown that such a strategy can produce a great bias in certain cases (e.g., simulations have shown that a Pearson's *r* of .8 could be observed under random mating in certain scenarios; see Rolán‐Alvarez et al., 2015), caused by the scale‐of‐choice effect (SCE). The concept of the SCE is that different variants of a given trait can be distributed nonrandomly across spatial and temporal scales, and hence, pooling of mating pairs from such heterogeneous samples may lead to “false‐positive” results (Rolán‐Alvarez et al., 2015). Indeed, mating pairs can be difficult to observe/score in the field and, because of this, researchers often pool these pairs over a geographic range or time series (e.g., Jiang et al., 2013). The SCE will, therefore, occur when two conditions are met: firstly that the organism looks for a mate at a smaller scale than the pooled scale and, secondly, that at the smaller scale there is some trait heterogeneity (Figure 1). These two conditions could be common in organisms which exhibit low adult mobility (Rolán‐Alvarez et al., 2015) and has already been demonstrated for one species with negative assortative mating (Rolán‐Alvarez et al., 2015) and two species with positive assortative mating (Ng, Williams, Davies, Stafford, & Rolán‐Alvarez, 2016).

The major focus of the present study was, therefore, to develop new estimators of mating preference by similarity that are less biased by the SCE (as compared to traditional approaches using Pearson's coefficient *r*) and hence provide a better linkage between theoretical and experimental estimates of mating preference. We use a modified Gaussian function from traditional theoretical models to simulate positive assortative mating and thus obtain a set of simulated mating pairs with an a priori‐controlled strength of preference. With such a collection of simulated mating pairs, we were then able to evaluate a posteriori different estimators of the (a priori) strength of mating preference. The simulations were derived under the effect of several factors (trait mean and variance, differences in trait between sexes, scale of the trait, etc.) in order to assess how robust the estimations were. A second round of simulations were also run to evaluate how the estimates behaved under scenarios affected by SCE (sensu Rolán‐Alvarez et al., 2015). Finally, we provide examples to demonstrate the application of the new estimators (as compared to the traditional method using Pearson's *r*) on empirical data for three marine snail species: *Littorina fabalis*,* Echinolittorina malaccana*, and *Echinolittorina radiata,* where the SCE has already been demonstrated (Ng et al., 2016; Rolán‐Alvarez et al., 2015).

## 2 Materials and Methods

### 2.1 Estimating mating preference

Several Gaussian mathematical functions have been used to infer mating preference under the similarity preference model (Carvajal‐Rodríguez & Rolán‐Alvarez, 2014; Débarre, 2012; Dieckmann & Doebeli, 1999; Gavrilets & Vose, 2007; Gavrilets, Vose, Barluenga, Salzburger, & Meyer, 2007; Thibert‐Plante & Gavrilets, 2013; Servedio, 2015). These functions predict the probability of mating for any particular pair based on a few key parameters (Gavrilets, 2004), namely: (1) the *C* parameter (equivalent to Pearson's *r* in empirical approaches) which represents the strength of mating preference for a trait which is supposedly evolving and contributing to assortative mating; (2) the *D* parameter, which represents the absolute difference between male and female trait values (see Equation (1) below). In addition, several of these functions include a parameter, *s* ^2^, which allows fine‐tuning of the preference under simulated conditions, but is assumed to be biologically irrelevant and is maintained constant within the simulation (Carvajal‐Rodríguez & Rolán‐Alvarez, 2014; but see an alternative strategy in Jennions & Petrie, 1997). Most theoretical functions were defined for a specific *D* scale (typically *D*_max_ = 1,), but we chose the function FND because it is scale independent and hence applicable to empirical data which may not fit well into the *D* = 1 scale (Carvajal‐Rodríguez & Rolán‐Alvarez, 2014). Under positive assortative mating (*C* > 0 parameter; see example below) the FND function value will be proportional to the probability of mating (*p*) for a given couple having certain trait values (*D* parameter).

(1) $$p\propto e^{\left(-C^{2}\times D^{2}\right)/\left(s^{2}\times D_{\max}^{2}\right)},$$

where *s* ^2^ is the mating tolerance, *C* is the mating preference itself (range from 0 to 1), *D* is the absolute difference between male (*X*_m_) and female (*X*_f_) unstandardized traits (size or shell length in this case) for each pair evaluated, and *D*_max_ is the maximum *D* value that can be observed in the population. For example, we can model positive size assortative mating (say *C* = 0.5) by computer simulation and obtain a series of *N* random male and female size pairs from a population (from certain a priori population mean and variance; see Table S1 and corresponding explanations in Appendix S1). Therefore, the encounter between a male and a female is random but whether they will mate or not depends on the mating probability given by the preference function FND. The FND value of each mating pair is calculated by Equation (1). Once we have the FND values of the *N* randomly formed couples, a Monte Carlo procedure based on pseudorandom numbers (as is the standard practice) will pick‐up the mating pairs so that the probability of being chosen is proportional to their FND values (see Appendix S1). The resulting set of mating pairs is expected to show a Pearson's *r* (for size) close to 0.5 (see Table S1). In this example, the preference parameter is *C* = 0.5, which has been established a priori, while the measured Pearson's *r* is a posteriori and could be considered as an estimate of the *C* parameter.

**Figure 2:**
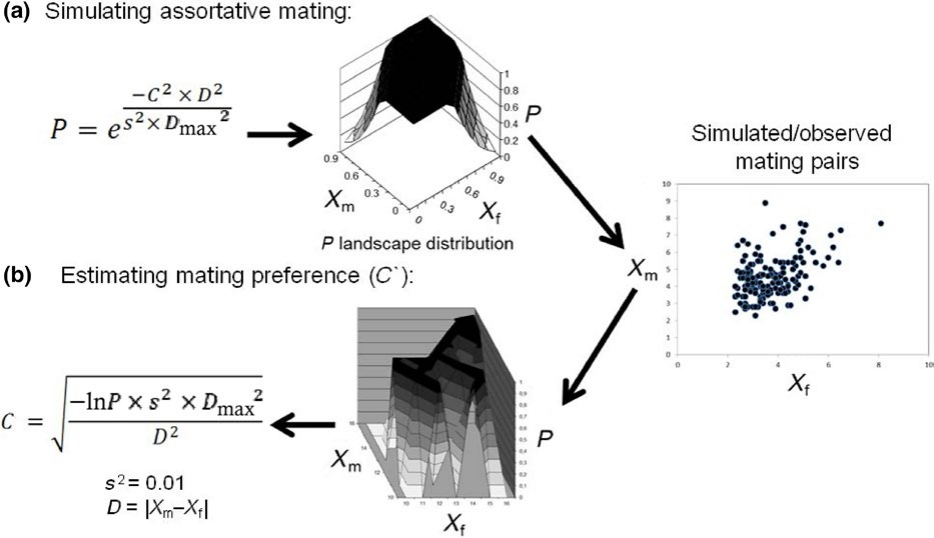
(a) Scheme of how the FND function can be used to simulate a set of mating pairs under certain a priori strength of mating preferences. (b) Scheme of how a set of observed/simulated mating pairs can be used to estimate any *C* parameter (*C*_scaled_ or *C*_rough_, see Section 2)

We were interested to check the robustness of the new mating preference estimators proposed in this study following a particular trait distribution in mating pairs under a positive assortative mating scenario (*C* > 0). To do this, we firstly used the Pearson's correlation coefficient *r* (the traditional approach for empirical data) and secondly two versions of a direct estimate of the *C* parameter (*C*_scaled_ and *C*_rough_) from the FND mating preference function. An illustration of how the mating pairs can be simulated by FND is shown in Figure 2a, while Figure 2b illustrates how the strength of the mating preference (both *C*_scaled_ and *C*_rough_) can be estimated from the observed/simulated pairs.

**Figure 3:**
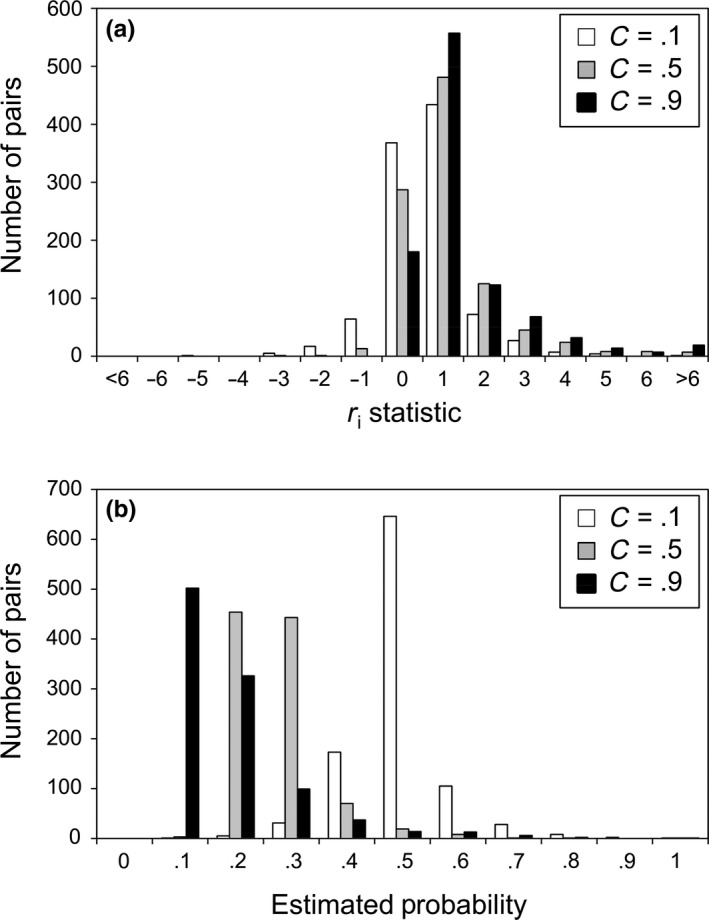
Example of conversion of *r*_i_ statistics to mating probabilities for a similar set of putative mating pairs obtained from a population with the same mean and variance and under different mating preferences (*C* values). (a) Histogram of *r*_i_ statistic (contribution of each pair to Pearson's *r*; see Perez‐Figueroa et al., 2008) generated in populations under different a priori strength of mating preferences (*C*). (b) The former *r*_i_ values were rescaled from 0.01 to 0.99 to match probability estimates. Note that, as expected, the mating pairs would produce a probability distribution with lower mean *P* under high preference (*C* = 0.5), because a high preference would render a high probability exclusively if the pairs show very similar trait values (rare cases with low *D*)

Given a sample of observed or simulated mating pairs, the algorithmic procedure to estimate *C*, by the positive assortative mating FND function, is as follows:

1. Calculating the *p* value of every copulating pair from the observed set of mating pairs in the studied population using the *r*_i_ statistic (where *r*_i_ = *Z*_m_ × *Z*_f_; as an estimate of the assortative mating for each pair separately; Perez‐Figueroa et al., 2008), where *Z*_m_ and *Z*_f_ are the male and female standardized traits (*X*_male_ and *X*_female_) values. The range of values observed for *r*_i_ in the population is rescaled (0.01–0.99) to avoid indeterminate solutions when estimating *C* from Equation (1) (see step 3 below and Figure 3 for an example of conversion of *r*_i_ to probabilities).
2. Estimating for every pair the value of *D* (*D* = |*X*_male_ − *X*_female_|) and *D*_max_ for each population (*D*_max_ = |*X*_max_ − *X*_min_|). *X* is the value of the trait (shell size in our experimental model) used in the pair (*X*_male_, *X*_female_) or in the population (*X*_min_, *X*_max_). The same tolerance is used in all simulations and during empirical estimation (*s* ^2^ = 0.01).
3. Solving *C* from Equation (1). This approach occasionally gives *C* estimates (*C*′) larger than 1, and so the way to correct for this will characterize the two alternative statistics proposed: *C*_rough_ excludes any *C* value larger than 1, and so the sample size for estimation would be reduced when the data sample size is low and the a priori *C* values high. Alternatively, *C*_scaled_ allows all *C* values, but the final mean estimate is rescaled to range between 0 and 1.

### 2.2 Validation of the estimation process by simulations (EP simulation)

Simulations were undertaken to validate the mating preference estimations under different scenarios (Table 1). Simulations were achieved by generating different mating pairs (*N*_pair_ = 30, 100 and 1,000) under different levels of positive assortative mating (*C* range 0–1, with 0.1 steps under FND). Additionally, the scenarios involved different *D*_max_ ranges (1, 5, and 10) and four different trait distributions (with different mean and variances across sexes: distribution 0–3 in Table 1). The null case distribution (case 0) considered certain mean (*D*_max_/2) and standard deviation (*D*_max_/4; named case 0), but considered three further alternative scenarios: case 1 (female mean = 5 × *D*_max_/2), case 2 (*SD *= *D*_max_), and case 3 (*SD*= *D*_max_; female mean= *D*_max_). Mating preferences were simulated using different tolerances (*s* ^2^= 0.1, 0.01, and 0.001, although as the results were qualitatively similar, only results for 0.01 are presented). Each simulation was repeated 1,000 times. Once the mating pairs were generated, mating preferences were estimated by using classical Pearson's correlation (*r*) and *C*′, as explained above.

**Table 1:** Combination of scenarios used in the two different simulations (estimation process [EP] and scale‐of‐choice effect [SCE])

Simulation	Choice	*N*_pair_	*D*_max_	Distribution	Ngroup	CV	*SD*	*N* Scenarios
EP	0–1 step 0.1	30, 100, 1,000	1, 5, 10	0, 1, 2, 3	—	—	—	396
SCE	0–1 step 0.1	20, 100, 500	1	0	10, 100	0–1 step 0.1	0.1, 0.3, 0.45	2,178

*N*_pair_ is number of pairs simulated, *D*_max_ the maximum possible difference in the population for the trait. Distribution represents four distinct scenarios for mean and variance of the trait across sexes. For the SCE simulation, *N*_group_ is the number of subgroups simulated, CV the coefficient of variation expected across the simulated subgroups and *SD* the standard deviation within those groups. Finally, *N* scenarios are the number of combinations of scenarios in each simulation. Each combination was replicated 1,000 times.

These estimates were compared with the a priori true *C* values and, therefore, the robustness of the different estimators (*r*,* C*_rough_, and *C*_scaled_) was compared by measuring bias (= true *C* − estimated *C*), range of estimation, regression coefficient between estimators and true *C*, and coefficient of variation among computer samplings (which allow inference of sampling robustness), as in Carvajal‐Rodríguez and Rolán‐Alvarez (2014).

### 2.3 Validation of the scale‐of‐choice effect by simulations (SCE simulation)

The SCE is the bias caused by measuring assortative mating at an inappropriate scale (Rolán‐Alvarez et al., 2015), and it can be measured by the difference between the estimator (e.g., *r* or *C*_rough_) at the incorrect scale—the estimator at the appropriate/true scale (Figure 1). In order to investigate how SCE could affect our estimators, an additional set of simulations were performed following the same scenarios used above (Table 1; Rolán‐Alvarez et al., 2015), using 11 choice (*C*) values, three different numbers of pairs (*N* = 20, 100 and 500), two sets of subgroups (*N*_groups_  = 10 and 100) to contribute to the SCE with 11 different levels of variation among those subgroups (coefficient of variation; CV = 0–1, step by 0.1), and three different levels of variation within groups (*SD* = 0.1, 0.3, and 0.45; Table 1). The SCE bias is expected to emerge whenever CV is larger than 0 as demonstrated by Rolán‐Alvarez et al. (2015) using Pearson's coefficient *r*. The new simulations introduce a few relevant differences in the method to simulate assortative mating as compared to the previous simulations. Specifically, while in Rolán‐Alvarez et al. (2015) mating pairs were generated from a correlated bivariate distribution (with a range of *C* from −1 to 1), in the present simulation we used the FND function to mimic the mating preference (range of *C* from 0 to 1, step by 0.1; Table 1).

### 2.4 Estimating mating preference from wild mating pair (empirical) data

The new estimators (*C*_rough_ and *C*_scaled_) were applied and compared with the classical Pearson's *r*, to mating pair data (shell size) from species where SCE has been previously detected (*L. fabalis*,* E. malaccana*, and *E. radiata*) using both published data on the two *Echinolittorina* species and unpublished data from *L. fabalis* (Ng et al., 2016; Rolán‐Alvarez et al., 2015). The locality and sampling details for *L. fabalis* were identical to the Rolán‐Alvarez et al. (2015) study except that the samples were obtained in July 2014. The SCE measures the magnitude of bias in estimating the correlation coefficient by taking into account the nonrandom distribution of different size classes among the samples from different small areas on the shore (see Figure 1). Five homogeneous sets of size classes (or subgroups) were used in the SCE analyses, derived from the mean individual size in each small area. The Statistics_averaged_ was, therefore, calculated over these homogeneous sets of size classes, and the significance of the SCE was evaluated by comparing the Statistics_averaged_ (Pearson's *r* or *C*_rough_) against the Statistics_pooled_ as a null value using a *t* test. The SCE can, therefore, be estimated as Statistic_pooled_ − Statistic_averaged_ across the five classes (see Ng et al., 2016; Rolán‐Alvarez et al., 2015). We also added a short simulation step by resampling the empirical data under *C* = 0 in order to statistically check whether the observed *C*_rough_ could be explained solely by random mating. The algorithm to calculate *C*_rough_ and Pearson's *r* from empirical data were implemented in C++, and the software is available from DRYAD (Fernández‐Meirama et al., 2017; https://doi.org/10.5061/dryad.5jd7j).

## 3 Results

### 3.1 Validation of estimation process

**Figure 4:**
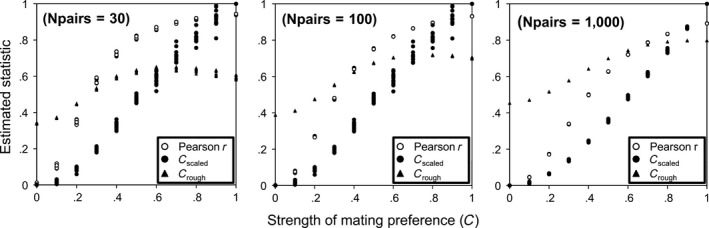
All estimated statistics (Pearson's *r*,* C*_scaled_, and *C*_rough_) for the true strength of the preference (*C*) simulated a priori and the three different mating pair sample sizes (*N*_pairs_). The true values regressed against the estimated statistics are shown. Note that the statistics are basically not affected by the six scenarios (*N*_group_ × *SD*; see Table 1) considered

The robustness of the three estimators of positive mating preference by similarity, *C*_rough_, *C*_scaled_, and Pearson's correlation coefficient (*r*) were evaluated (Table 2). All statistics showed a high and significant (all cases *p* < .05) linear regression slope, but only Pearson's *r* and *C*_scaled_ showed a slope close to 1 (Table 2), and hence, these two estimators of mating preference (*C*) were relatively more robust than the *C*_rough_ considering this property (Figure 4). This *C*_rough_ limitation in estimating *C* occurs because the range of the estimated values was only about one‐third of expected values (Table 2, Figure 4). The robustness of all estimators was improved with larger sample sizes (*N*_pair_; see Table 2). Additionally, the overall error in estimation of *C* was relatively moderate for the three estimators (expected mean value should be 0.5), although the bias for the *C*_rough_ and *C*_scaled_ increased somewhat at the largest sample size (Table 2). These properties were rather insensitive to the different scenarios proposed (see low *SD* in Table 2), and the estimation errors within each scenario were typically small enough to effectively distinguish the *C* values differing by 0.1 units (except for *C*_rough_ when estimating values of *C* larger than 0.6, Figure 4). When using simulation averages across scenarios, Pearson's *r* and *C*_scaled_ outperformed *C*_rough_ in estimating *C*. The sampling robustness of estimators was measured by the mean coefficient of variation of the different statistics across the 1,000 computer simulations within the scenarios (summarized across scenarios by averages ± *SD*): CV_Pearsonr_ = 1.0% ± 1.5; CV_Cscaled_ = 392% ± 818.0; CV_Crough_ = 9% ± 1.3. The results clearly showed that both Pearson's *r* and *C*_rough_ outperformed *C*_scaled_, which showed severe sampling errors during simulations, which limits the utility of this estimator.

**Table 2:** Summary of results obtained under the estimation process simulation for the three statistics (Pearson's *r*,* C*_scaled_, and *C*_rough_)

*N*_pair_	*D*_max_	Distr.	Pearson's *r*		*C*_scaled_		*C*_rough_	
			Slope	Mean	Slope	Mean	Slope	Mean
30	1	0	0.96	0.64	1.07	0.46	0.26	0.54
		1	0.96	0.64	1.09	0.48	0.28	0.55
		2	0.95	0.65	1.07	0.46	0.30	0.56
		3	0.96	0.65	1.09	0.46	0.30	0.56
	5	0	0.96	0.64	1.09	0.46	0.27	0.54
		1	0.96	0.64	1.04	0.44	0.28	0.55
		2	0.95	0.66	1.12	0.47	0.30	0.56
		3	0.95	0.65	1.11	0.47	0.30	0.56
	10	0	0.96	0.64	1.12	0.48	0.26	0.54
		1	0.96	0.64	1.12	0.46	0.28	0.55
		2	0.95	0.65	1.09	0.46	0.29	0.55
		3	0.95	0.65	1.15	0.49	0.29	0.55
Averaged ± *SD*			0.95 ± 0.005	0.65 ± 0.007	1.10 ± 0.031	0.47 ± 0.013	0.28 ± 0.014	0.55 ± 0.005
100	1	0	0.99	0.61	1.08	0.44	0.36	0.61
		1	0.99	0.60	1.08	0.45	0.36	0.61
		2	0.99	0.60	1.08	0.44	0.36	0.61
		3	0.99	0.60	1.08	0.45	0.36	0.61
	5	0	0.99	0.60	1.05	0.43	0.35	0.61
		1	0.99	0.60	1.05	0.43	0.35	0.61
		2	0.99	0.60	1.04	0.43	0.36	0.61
		3	0.98	0.61	1.08	0.44	0.36	0.61
	10	0	0.99	0.60	1.07	0.42	0.36	0.61
		1	0.98	0.61	1.08	0.43	0.36	0.61
		2	0.99	0.61	1.08	0.43	0.36	0.61
		3	0.99	0.61	1.04	0.42	0.36	0.61
Averaged ± *SD*			0.99 ± 0.001	0.60 ± 0.001	1.07 ± 0.018	0.43 ± 0.010	0.35 ± 0.023	0.61 ± 0.001
1,000	1	0	0.99	0.53	1.06	0.42	0.40	0.66
		1	0.99	0.53	1.06	0.42	0.40	0.66
		2	0.99	0.53	1.06	0.42	0.40	0.66
		3	0.99	0.53	1.06	0.42	0.40	0.66
	5	0	0.99	0.53	1.05	0.41	0.39	0.66
		1	0.99	0.53	1.06	0.41	0.40	0.66
		2	0.99	0.53	1.06	0.41	0.39	0.66
		3	0.99	0.53	1.06	0.41	0.39	0.66
	10	0	0.99	0.53	1.06	0.41	0.40	0.66
		1	0.99	0.53	1.05	0.41	0.40	0.66
		2	0.99	0.53	1.06	0.41	0.40	0.66
		3	0.99	0.53	1.06	0.41	0.40	0.66
Averaged ± *SD*			0.99 ± 0.001	0.53 ± 0.000	1.06 ± 0.004	0.41 ± 0.003	0.40 ± 0.001	0.66 ± 0.001

*N*_pair_, *D*_max_, and Distribution (Distr.) as in Table 1. The regression coefficient b of the true choice simulated against the estimate (Slope) is given, as well as the mean of the estimates across the full set of choices simulated (expected value 0.5).

### 3.2 Validation of SCE

**Figure 5:**
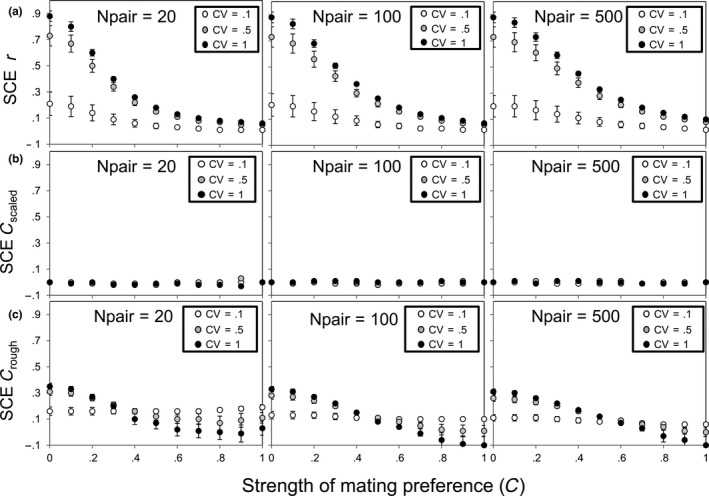
Representation of the magnitude of the mean simulated scale‐of‐choice effect error (Statistic_pooled_ − Statistic_averaged_; with corresponding standard errors) for the different estimators of mating preference at different simulated preference strengths (*C*): (a) Pearson's *r*, (b) *C*_scaled_, and (c) *C*_rough_. The relationship is summarized for three representative coefficients of variation (CV) and three mating pair sample sizes (*N*_pair_)

The sensitivity of each estimator (Pearson's *r*,* C*_scaled_, and *C*_rough_) of mating preference by similarity to the SCE bias was evaluated (Tables 1 and 3). The results were averaged across subgroups (CV) and level of variation within groups (*SD*) as they did not produce any great variation on SCE trends (except under small CV; see Figure 5). Pearson's *r*, as expected, showed a strong bias for those scenarios that included the pooling of subgroups which showed a certain degree of heterogeneity (i.e., CV > 0.5). The bias was rather insensitive to sample size (*N*_pair_; Table 3). The SCE biased the estimation of mating preference (*C*) based on Pearson's *r* from low to high values (up to 0.6), while *C* estimates based on *C*_rough_ and *C*_scaled_ were biased to a much lesser extent (moderately to no bias; Figure 5). In this case, *C*_scaled_ and *C*_rough_ clearly outperformed Pearson's *r* and were less sensitive to the problems associated with the SCE.

**Table 3:** Summary of the mean scale‐of‐choice effect (SCE) bias (statistic_pooled_ − statistic_averaged_; see Section 2) obtained under the SCE simulation for the three statistics (Pearson's *r*,* C*_scaled_, and *C*_rough_) for all *N*_pair_ and choice values and averaged across the rest of factors (Ngroups, *SD* and CV)

*N*_pair_	Choice	SCE		
		Pearson's *r*	*C*_scaled_	*C*_rough_
20	0	0.62	0	0.28
	0.1	0.56	0	0.27
	0.2	0.42	−0.01	0.23
	0.3	0.28	−0.01	0.19
	0.4	0.18	−0.01	0.15
	0.5	0.13	−0.01	0.12
	0.6	0.09	−0.01	0.09
	0.7	0.07	−0.01	0.09
	0.8	0.05	0	0.08
	0.9	0.05	0	0.09
	1	0.04	0	0.11
	Averaged	0.23 ± 0.214	−0.01 ± 0.005	0.15 ± 0.076
100	0	0.62	0	0.25
	0.1	0.58	0	0.24
	0.2	0.48	0	0.22
	0.3	0.36	0	0.18
	0.4	0.26	−0.01	0.14
	0.5	0.18	−0.01	0.1
	0.6	0.13	−0.01	0.07
	0.7	0.1	−0.01	0.05
	0.8	0.08	−0.01	0.02
	0.9	0.06	0	0
	1	0.05	0	0
		0.26 ± 0.213	0.00 ± 0.005	0.12 ± 0.096
500	0	0.62	0	0.23
	0.1	0.59	0	0.23
	0.2	0.52	0	0.21
	0.3	0.42	0	0.18
	0.4	0.32	0	0.14
	0.5	0.24	0	0.11
	0.6	0.18	0	0.08
	0.7	0.13	−0.01	0.05
	0.8	0.1	0	0.03
	0.9	0.08	0	0.01
	1	0.07	0	−0.01
		0.30 ± 0.209	0.00 ± 0.003	0.11 ± 0.089

### 3.3 Application of the new estimators of mating preference to empirical data

The estimations of mating preference (*C*) using *C*_scaled_ were too noisy to be useful (see above) and are not presented, but the estimated *C* based on *C*_rough_ averaged across the five homogeneous subgroups and its corresponding estimated SCE are illustrated in Table 4. The *C*_rough_ across samples was relatively similar between species (around 0.4). The estimated SCE was, however, reduced by half in *E. malaccana* and *E. radiata,* although it remained similar in *L. fabalis,* which indicates the ability of *C*_rough_ to reduce the SCE bias at least in those cases with the highest SCE.

**Table 4:** *C*_rough_ estimates from experimental data, and experimental estimations of the scale‐of‐choice effect (SCE) for this new estimator, which can be compared with the SCE estimates from Pearson's *r*

Species	Locality	Pearson's *r*	All samples		*C*_averaged_	SCE_C_
		SCE_5_^®^	*N*	*C*_pooled_		
*Echinolittorina malaccana*	Shek O	0.49*	40	0.51	0.32 ± 0.051	0.19**
	Cape D'Aguilar	0.47*	228	0.60	0.38 ± 0.025	0.22**
*Echinolittorina radiata*	Cape D'Aguilar	0.54*	49	0.53	0.30 ± 0.041	0.23***
*Littorina fabalis*	Abelleira	0.12	95	0.40	0.28 ± 0.044	0.13***

The SCE is experimentally obtained by *C*_pooled_ − *C*_averaged_ (see Ng et al., 2016; Rolán‐Alvarez et al., 2015).

*p < .05; **p < .01; ***p < .001.

## 4 Discussion

A mathematical description of any potential evolutionary mechanism is a prerequisite to fully understand and predict biological phenomenon (Servedio et al., 2014). In this study, we proposed a new method to estimate positive mating preference by similarity using the FND mathematical function (Carvajal‐Rodríguez & Rolán‐Alvarez, 2014). This strategy can be used to infer mating preference in organisms that show positive assortative mating for size (or any similar trait in both sexes). The method is based on the assumption that, without a priori knowledge of the genetic mechanisms contributing to the preference, a mathematical function can amalgamate all the preferences into one variable, *C* (sensu Gavrilets, 2004, 2014; Thibert‐Plante & Hendry, 2011; Débarre, 2012; Thibert‐Plante & Gavrilets, 2013; Roff & Fairbairn, 2015), which itself could be determined by many quantitative loci. Such a strategy has been used since the origin of quantitative genetics (Falconer & Mackay, 1996) but previously was only used for making theoretical predictions. The FND Gaussian‐like function is a modification of the traditional methods used in theoretical studies which is able to accommodate empirical data and as such provides a link between the two research approaches.

For the first time, we were able to formally evaluate how the classical Pearson's *r* is related to the strength of mating preference using the FND function in a combination of simulations and empirical data. Interestingly, Pearson's *r* showed excellent estimation properties and allowed efficient estimations of mating preferences in all scenarios, except in situations when the SCE was simulated. Here we showed that when SCE was not present, Pearson's *r* could be a valuable tool to estimate the strength of mating preference, but as shown previously (Rolán‐Alvarez et al., 2015), when the SCE is present, it can produce huge bias in Pearson's *r* as an estimator of mating preference. Therefore, for any model organism in which SCE has been experimentally shown to be small or negligible, Pearson's *r* can be used to infer mating preference directly in the wild. Using such an approach, theoretical predictions and empirical studies can be connected, which allows fundamental progress in our understanding of the role of mating preference in driving genetic differentiation in the wild (Gavrilets, 2004; Roff & Fairbairn, 2015; Servedio, 2016). Future theoretical predictions regarding mating preferences by similarity can, therefore, be empirically verified whenever the study has corrected for any potential SCE bias. Where there is a bias due to the SCE, there are only two known strategies to correct for this. The first uses the information of nonmating individuals surrounding the mating pair to reorganize the dataset into a series of homogeneous subgroups and then uses the averaged of Pearson's *r* across subgroups to correct for the pooled estimate (see Figure 1 and Table 4; also see Rolán‐Alvarez et al., 2015; Ng et al., 2016). This strategy is feasible, but it requires extra sampling effort and cannot be used on published data that have not applied an appropriate experimental design.

The second strategy makes use of specific estimators of the strength of mating preference, such as the *C*_scaled_ or *C*_rough_ described here. From our evaluation of the two new estimators (*C*_rough_ and *C*_scaled_), one of them (*C*_scaled_) showed ideal theoretical properties but failed when applied to realistic sample sizes, while the other (*C*_rough_) showed limited theoretical properties but behaved reasonably well at low sample sizes. We also empirically demonstrated that *C*_rough_ greatly reduced the SCE bias as compared with the traditional approach using Pearson's *r* in some cases (*C* < 0.6). This new estimator, therefore, could be useful and provide a complementary approach with Pearson's *r* (showing high bias due to SCE for *C* < 0.5) to infer mating preferences directly in the wild. The theoretical limitations of *C*_rough_, however, suggest it should be used with caution, especially when the estimate shows values larger than 0.60, as such values are not proportional to the true strength of mating preference (Figure 4). Comparing field data with how the different estimators behave further corroborated the simulated results. *C*_rough_ statistics could reduce (even half) the SCE effects compared to Pearson's *r*. The *C*_rough_ statistics, therefore, can be applied to those datasets which lack information about nonmating individuals surrounding the mating pair in order to check whether such estimators do, in fact, change any interpretation based on Pearson's *r*. It would be insightful, for example, to reanalyze the data reviewed by Jiang et al. (2013) to see whether halving the SCE bias on average changes the overall patterns observed.

A new question that arises is why our new estimators seem to be less sensitive to issues of the SCE or why Pearson's *r* coefficient is more sensitive to the SCE. In fact, statisticians have yet to have a good understanding of why Pearson's *r* coefficient is affected by data heterogeneity producing such unpredictable biases (see discussion in Hassler & Thadewald, 2003). The new proposed estimators are based on a different algorithm from Pearson's *r* which is known to be extremely affected by outliers (Rousselet & Pernet, 2012). In addition, our methods indirectly limit the effects of outliers due to partial rescaling (or excluding extreme values), and this could be part of the explanation. Nevertheless, more research will be needed to understand this kind of bias (or its absence) in statistics related either directly or indirectly to correlation coefficients. The new proposed estimators could, however, be further improved in the future, ideally to a level without bias due to the SCE in estimating mating preference.

Several authors have called for improvement in the relationship between theoretical and empirical methodologies to allow progress in evolutionary theory (Gavrilets, 2004, 2014; Roff & Fairbairn, 2015; Servedio, 2015). In this paper, we add to the strategy initiated by Roff and Fairbairn (2015) trying to connect both frameworks, by proposing a new estimator (*C*_rough_) for mating preferences (as well as checking the applicability of Pearson's *r* for the same purpose) from mating pairs directly captured in the wild. Although the method could be problematic for estimating unbiased preferences, it may be sound and robust enough for comparing estimates among groups and testing hypotheses on mate choice evolution. The priority would be to use this function in theoretical and empirical studies, as well to check whether theoretical predictions can be supported or rejected by observations in the field. Our approach could be applied, for example, to ecological models for studies of speciation, such as *Littorina saxatilis* (Rolán‐Alvarez, 2007), stick‐insects (Nosil, Egan, & Funk, 2008; Nosil & Feder, 2013), the stickleback (Kraak & Hart, 2011; Hendry, Hudson, Walker, Räsänen, & Chapman, 2011; Vines et al., 2016), or cichlids (Gavrilets et al., 2007; Martin, 2013; Seehausen et al., 2008), to check whether theoretical predictions match empirical estimates in the wild. Additionally, this methodology could be used for testing whether runaway sexual selection could contribute to the allopatric process of speciation (reviewed in Servedio & Bürger, 2014).

## Conflict of Interest

The authors do not have any conflict of interest.
