# Supplementary material for: A novel method for estimating the strength of positive mating preference by similarity in the wild
Source: Ecol Evol. 2017 Mar 22;7(9):2883–93. doi: 10.1002/ece3.2835 (PMC5415541; doi:10.1002/ece3.2835)
Supplement: Supplementary file 1 [file ECE3-7-2883-s001.docx]

**Appendix section from**

**A novel method for estimating the strength of positive mating preference by similarity in the wild**

Mónica Fernández-Meirama^1^, Daniel Estévez^1^, Terence P.T. Ng^2^, Gray A. Williams^2^, Antonio Carvajal-Rodríguez^1^ and Emilio Rolán-Alvarez^1^

*1 Departamento de Bioquímica, Genética e Inmunología, Facultad de Biología, Universidad de Vigo, 36310 Vigo, Spain*

*2 The Swire Institute of Marine Science and School of Biological Sciences, The University of Hong Kong, Pokfulam Road, Hong Kong SAR, China*

RUNTITLE: ESTIMATING MATING PREFERENCE IN THE WILD

Appendix: Simulation of matings with the FND preference function

In this appendix we explain in detail how the FND (and other Gaussian functions; see Carvajal-Rodríguez and Rolán-Alvarez 2014) can be used to simulate assortative mating. We plan to simulate the extraction of 20 mating pairs (in 3 replicates; see Table A1) from a particular population with moderate mating preference by similarity (C = 0.5 using the FND function; see M&M) for a particular trait (mean ± SD = 10 ± 5). The software chooses a random set of 20 male and female. The “Male” column in Table 1A represents the 20 males and the "Potential" column represents the 20 females chosen *a priori* for passing through the mating selection process. Thus, performed in this way the encounter between a male and a female occurs at random but whether they will mate or not depends on the mating probability as obtained from the preference function. Under our model every male mates but the females can mate from none to several times depending on the male choosing ability. Now a standard Monte Carlo process is applied as follows. Let’s consider male 2 from CASE 1 (see Table A1) as a numerical example. For any given male, say male *i*, we compute all the FNDs that result from the potential mate of such male with the 20 available females, in that case male 2 has a FND of 0.398317 with female 1. The FND of the same male with female 2 is 0.00015535, with female 3 is 0.467283 and so on... The 20 FNDs obtained (one for each female) are summed so that the mating probabilities for this male are obtained by dividing the FND values by the total sum. So we finally have a set of 20 mating probabilities (summing up to 1) for the male *i*., The sum of the 20 FNDs for potential couples with male 2 is 6.30633007 and therefore the probability is obtained dividing each FND by this total, being correspondingly (P_1_=0.06316146; P_2_= 2.46x10^-5^ and P_3_=0.07409745, etc). The mating distribution function for this male is obtained by accumulating the probability values so that F(i,1) = 0.06316146, F(i,2) =0.06316146+2.4634x10^-5^=0.06318609, F(i,3) =0.06318609+0.07409745= 0.13728355 and so until the last female that will be F(i,20) = 1 (see a pie chart representation of this distribution for this particular example in Figure A1). We now obtain a random number between 0 and 1 say U = 0.1578, and as F(i,3) < U ≤ F(i,4), then the female 4 was chosen (with a trait value of 6.84; see Table A1 and Figure A1). Because it is assumed that all the males mate, this process is repeated for every male in the sample.

This example shows the key difference between the *a priori* simulated mating preference (C=0.5) and the *posteriori* one estimated from empirical coefficients (Pearson r coefficient and C_rough_; see M&M). Let us explain how we do the estimation process using the same example as above. First, we obtain the r coefficient calculated *a posteriori* on the mating pairs chosen as described above(r = 0.847 in CASE 1; Table A1). Second, we calculate the r_i_ coefficient (*sensu* Perez-Figueroa et al. 2008) for each pair (see corresponding column in Table A1) to be used to estimate the probability of this couple (r_i_ needs to be rescaled from 0.01 to 0.99 to convert to probabilities; see M&M). Then we can use FND algorithm to obtain the C estimate (using male-female trait difference as D=1.16 in this example; and s^2^=0.01 as a constant). The average of the C_rough_ across pairs is the proper estimate (C_rough_=0.590 in CASE=1; Table A1). Note that only C_rough_ estimates from 0 to 1 values were included in the averages (see N_Crough_ in Table A1). Therefore, both r coefficient (0.847) and C_rough_ (0.590) statistics do represent a estimation of the *a priori* C=0.5 value. Our simulations do repeat the same process for different scenarios and level of mating preference and using 1000 replications.

Table A1. Process of choosing 20 mating pairs from in a particular population with 3 replications (case 1-3; see text). The population had a mean trait value of 10 (SD=5). The true mate choice considered *a priori* (C=0.5) as well as the *posteriori* estimated coefficients (Pearson r and C_rough_) are shown below for every case. N_Crough_ is the number of pairs used for C_rough_ estimation (see text). The bold cases of CASE=1 are used as an example in Figure A1.

Figure A1. Different coloured portions of the wheel represent accumulative probabilities of the male 2 choosing the 20 potential females in CASE=1 (data from Table A1). Then a random number (U) is generated based the probabilities and it falls within one of the colour representing the chosen female. In this particular example, the female number 4 has been chosen (Trait value= 6.84; see Table A1).

Table A1:

|  | CASE 1 | | | |  | CASE 2 | | | |  | CASE 3 | | | |
| --- | --- | --- | --- | --- | --- | --- | --- | --- | --- | --- | --- | --- | --- | --- |
|  |  | Female | |  |  |  | Female | |  |  |  | Female | |  |
| Statistic | Male | Potential | Chosen | ri |  | Male | Potential | Chosen | ri |  | Male | Potential | Chosen | ri |
|  | 5.65 | 10.81 | 7.32 | 0.311 |  | 16.99 | 22.19 | 12.22 | -0.018 |  | 13.71 | -8.62 | 9.01 | -0.048 |
|  | **8.00** | 16.68 | **6.84** | 0.037 |  | 16.56 | 8.90 | 4.43 | -0.467 |  | 10.10 | 10.70 | 1.94 | -0.142 |
|  | 10.71 | 10.55 | 12.65 | 0.577 |  | 20.24 | 24.32 | 24.32 | 1.158 |  | -2.72 | 30.32 | 6.26 | 0.6379 |
|  | 3.68 | **6.84** | 3.40 | 1.734 |  | 6.77 | 9.90 | 12.22 | 0.014 |  | 7.37 | 6.16 | 10.70 | -0.027 |
|  | 14.05 | 13.28 | 13.28 | 1.603 |  | 26.99 | 7.77 | 24.32 | 2.026 |  | -0.56 | 19.09 | 1.94 | 1.184 |
|  | 12.72 | 9.19 | 13.07 | 1.177 |  | 1.80 | 4.43 | 9.90 | 0.268 |  | -1.77 | 4.95 | 6.26 | 0.5856 |
|  | 8.92 | 13.71 | 13.73 | 0.208 |  | 10.96 | 24.22 | 8.90 | 0.011 |  | 3.42 | 3.57 | 13.60 | -0.355 |
|  | 9.79 | 14.85 | 13.73 | 0.474 |  | 1.71 | 12.22 | 1.68 | 1.124 |  | 18.02 | -0.21 | 13.60 | 0.5799 |
| Preferred | 13.80 | 3.40 | 13.71 | 1.695 |  | 2.11 | -0.16 | 7.77 | 0.471 |  | -12.18 | 9.01 | 0.68 | 3.0593 |
| Trait | 9.81 | 7.32 | 7.44 | -0.176 |  | 13.86 | 4.43 | 21.89 | 0.267 |  | 7.23 | 0.68 | 10.70 | -0.03 |
|  | 7.56 | 16.43 | 7.32 | 0.082 |  | 7.22 | 21.89 | 1.68 | 0.475 |  | 21.39 | 17.21 | 6.16 | -0.698 |
|  | 2.17 | 13.81 | 5.11 | 1.618 |  | -9.68 | 10.40 | -0.16 | 2.886 |  | 16.24 | 8.41 | 9.01 | -0.074 |
|  | 11.46 | 5.11 | 7.44 | -0.360 |  | 16.97 | 1.68 | 24.22 | 0.730 |  | 10.85 | 6.11 | 9.01 | -0.019 |
|  | 2.41 | 13.73 | 3.79 | 2.065 |  | 16.47 | 26.94 | 24.32 | 0.672 |  | 10.45 | 1.94 | 6.94 | -0.065 |
|  | 7.54 | 16.83 | 10.81 | -0.078 |  | 20.75 | -1.29 | 28.55 | 1.661 |  | 21.36 | 13.60 | 17.81 | 1.6352 |
|  | 11.93 | 13.07 | 14.85 | 1.401 |  | 8.52 | 0.59 | 8.90 | 0.107 |  | 14.32 | 17.81 | 22.09 | 1.0772 |
|  | 6.32 | 7.44 | 6.84 | 0.290 |  | 19.22 | 6.56 | 7.77 | -0.411 |  | 15.95 | 22.09 | 6.94 | -0.304 |
|  | 3.55 | 8.12 | 3.40 | 1.785 |  | 21.73 | -9.60 | 21.89 | 1.071 |  | 23.21 | 18.82 | 19.09 | 2.1749 |
|  | 10.61 | 3.79 | 12.65 | 0.554 |  | -5.20 | 28.55 | -1.29 | 2.470 |  | -7.83 | 6.26 | 1.94 | 2.0866 |
|  | 4.12 | 12.65 | 5.11 | 1.100 |  | 10.91 | -1.97 | 6.56 | 0.022 |  | 10.65 | 6.94 | 19.09 | 0.2574 |
| C |  | 0.5 |  |  |  |  | 0.5 |  |  |  |  | 0.5 |  |  |
| Pearson r |  | 0.847 |  |  |  |  | 0.567 |  |  |  |  | 0.442 |  |  |
| C_rough_ |  | 0.590 |  |  |  |  | 0.607 |  |  |  |  | 0.455 |  |  |
| N_Crough_ |  | 12 |  |  |  |  | 11 |  |  |  |  | 13 |  |  |

Figure #A1
