# Supplementary figures and images for: A novel method for estimating the strength of positive mating preference by similarity in the wild
Source: Ecol Evol. 2017 Mar 22;7(9):2883–93. doi: 10.1002/ece3.2835 (PMC5415541; doi:10.1002/ece3.2835)

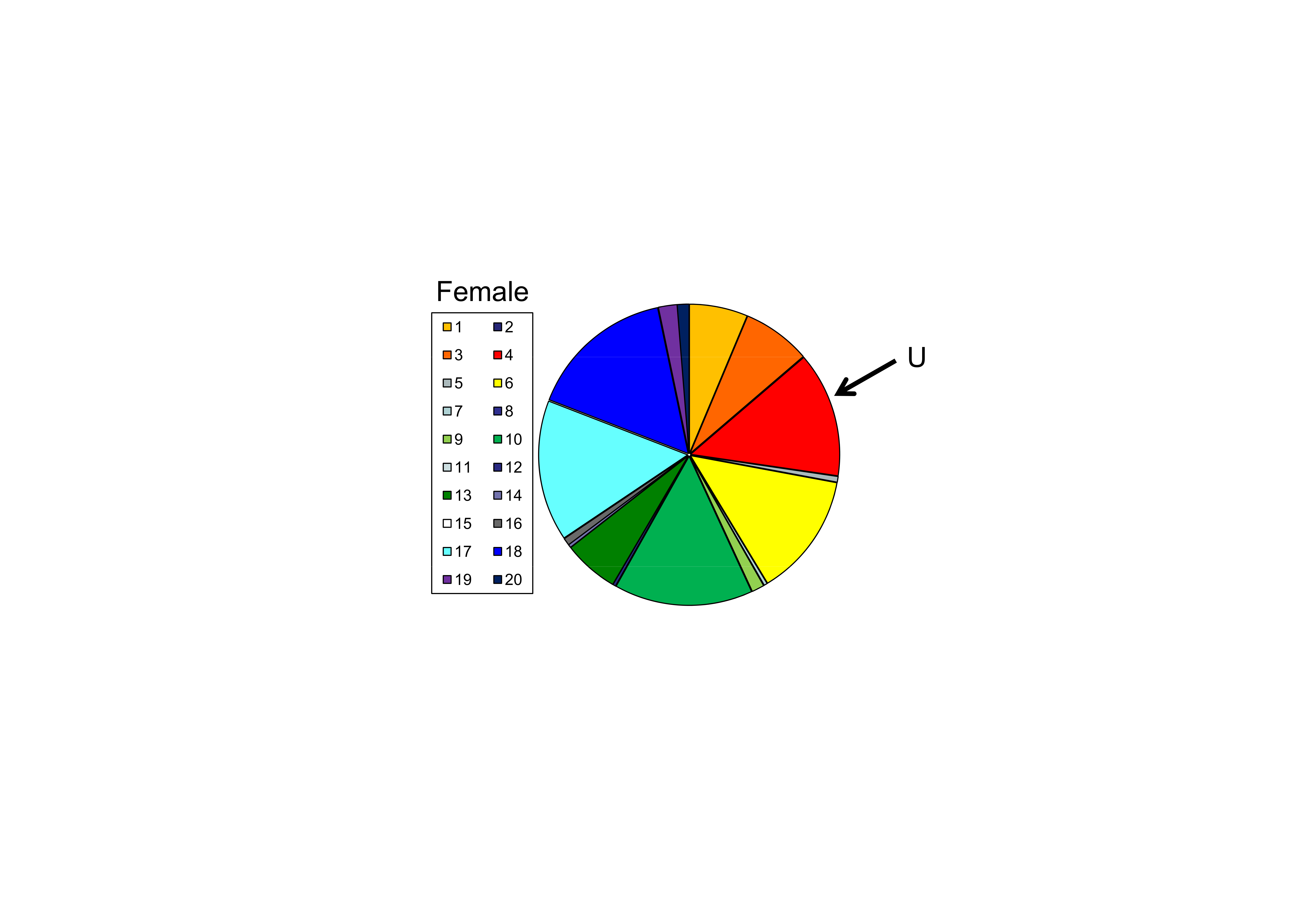

Supplement: Supplementary file 2 [file ECE3-7-2883-s002.tif]
